# Paragraphs of Interest

**Published:** 1893-05

**Authors:** 


### Editorial Department.

F. E. DANIEL, M. D., Editor.
S. E. HUDSON, M. D., Managing Editor.
A. J. SMITH. M. D., Galveston, Associate Editor.

EDITORIAL STAFF:

PROF. J. E. THOMPSON, M. D., Texas Medical College, Galveston; Surgery.
PROF. WM. KEILLER, M. D., Texas Medical College, Galveston; Obstetrics and Gynecology.
PROF. DAVID CERNA, M. D., Texas Medical College, Galveston; Therapeutics.
PROF. A. J. SMITH, M. D., Texas Medical College, Galveston; Medicine.
DR. ISADORE DYER, Tulane University; Dermatology.
DR. R. H. L. BIBB, Saltillo, Mexico; Foreign Correspondent.
DR. ROBT. MORRIS, Charity Hospital, N. O.; Clinical Reports.

The JOURNAL is the official organ of the Austin District Medical Society, the Wes Texas District Medical Society and the Galveston County Medical Society.

The Texas State Homeopathic Society *was to have met* at San Antonio on the 9th instant, but members did not materialize. Same thing happened last year, when meeting was called for Galveston. Alas! poor Yorick. When Fisher left Texas he carried "The Texas State Homeopathic Society" in his pocket; he was head, tail and body of the layout.

---

**State Medicine.**—It was a deserved compliment to our esteemed contemporary of the *Texas Sanitarian*, and a graceful recognition of his labors, to elect its editor, Dr. T. J. Bennett chairman of the Section on State Medicine and Public Hygiene. The *Texas Sanitarian* is a "clean" journal of sanitation. That may sound like a paradox, but it will be understood.

---

**The Cisco Cyclone.**—The friends of Dr. S. H. Stout throughout the South will rejoice to learn that the distinguished veteran surgeon and teacher was spared in the terrible visitation which destroyed Cisco. The Doctor writes in reply to an anxious inquiry: "Thank God, none of my family received any injury; a part of my residence was moved from its foundation. My loss is small." Not a church, school-building or hotel survived the crash.

---

The Legislature of the great State of Ohio has proved itself the highly representative body it has always been held to be, by enacting a law for the regulation of the practice of medicine and for requiring an examination before any man or woman shall be permitted to enter into practice of medicine—*upon horses.* The same high-minded body which thus distinguished itself refused even to give a decent hearing to a bill regulating the practice of medicine among human beings.

---

According to Dr. Jones, of New Orleans, during a period of thirty-eight years (from 1842 to 1880) 19,233 cases of yellow fever were treated in the Charity Hospital of that city, of which 9,667 (50.2 per cent.) terminated fatally. From 1844 to 1880 a total of 304,213 cases of various fevers, including yellow fever, were treated at the same institution, with a total of 43,718 deaths. Throughout the whole city during the same period there were 28,739 deaths from yellow fever, but during the same period there were also 24,071 deaths from pulmonary tuberculosis, and a grand total of 242,426 deaths from all causes.

---

Roll of Honor, Tex. Med. College.—First year students; the two sons of Dr. T. D. Wooten (President Board of Regents, University of Texas), Joe. Wooten and G. H. Wooten, are two of five who took first honors, by competitive examination, in anatomy; of four in physiology; of three in each, materia medica and pathology. Second year; J. H. Sampson took first honor in anatomy, physiology, surgery, obstetrics, pathology, histology and materia medica, and won the histological prize. In physiology, honors were divided with Duggen and Thompson.

Third year; T. T. Jackson took first honor in surgery, medicine and therapeutics, and won all three medals, one given by the State Medical Association, one by Dr. Keller, of Arkansas, and one by the College Faculty. The JOURNAL congratulates these young gentlemen on this great distinction.

---

The Commencement Exercises of the Texas Medical College (Medical Department University Texas) were held in Harmony Hall, Galveston, May 2. A vast audience of Galveston's *elite* graced the occasion, and the interesting event occurring during the time of the meeting of the Texas State Medical Association, a large number of medical men were present. There were two graduates—Drs. T. Terrell Jackson and Wm. Gammon. Their diplomas were presented by Hon. T. D. Wooten, M. D., President Board of Regents. Prof. Paine, Dean of the College, delivered the annual address and presented the medals. We regret that on account of our very full report of the Texas State Medical Association which appears in this issue, we are compelled to omit our correspondent's very interesting report of the Commencement, and the many pleasant and important events connected therewith, as well as all mention of the social and other incidental features of these two occasions. Dr. Gammon received his diploma by proxy, he having gone to Philadelphia to compete for an Interneship in a hospital.

